# Numerical simulation and tool parameters optimization of aluminum alloy transmission intermediate shell

**DOI:** 10.1038/s41598-024-54552-5

**Published:** 2024-02-20

**Authors:** Haiyue Zhao, Yan Cao, Yu Bai, Hui Yao, Chunlei Tian

**Affiliations:** 1 School of Mechatronic Engineering, Xi’an Technological University, No.2 Xuefu Middle Street, Weiyang District, Xi’an, 710021 China; 2 School of Computer Science and Engineering, Xi’an Technological University, No.2 Xuefu Middle Street, Weiyang District, Xi’an, 710021 China

**Keywords:** Thin-walled parts shell, Numerical simulation, Tool parameters optimization, Cutting experiment, Firefly algorithm, Aerospace engineering, Mechanical engineering, Computer science

## Abstract

Due to its challenging manufacturing and intricate morphology, the aluminum alloy transmission intermediate shell used in vehicle transmission has been the focus of many academic studies. In this study, the three-dimensional cutting model is condensed to a two-dimensional cutting model and utilized to simulate the finishing process of an aluminum alloy workpiece using the finite element modeling program DEFORM-3D. Through orthogonal testing and range analysis, the impact of integral end mill side edge parameters on cutting performance was investigated. It is determined that tool chamfering has a greater impact on cutting performance than tool rake and relief angles, that chamfering width has the most impact on cutting force, and that chamfering angle has the greatest impact on cutting temperature. The workpiece's surface roughness is tested during a cutting experiment, and an analysis of the data reveals that the finite element simulation model is accurate and the orthogonal test method is reasonable. The tool chamfer has a greater impact on roughness than the tool rake angle and relief angle. The tool settings are further optimized using the firefly method. By examining the data, it is determined that the prediction model is correct and the optimization model is reasonable. The cutting efficiency is higher and the surface quality is better when the chamfer width is 0.17 mm and the chamfer angle is 7.3° or 18.3°. Therefore, optimizing the side edge parameters of the integral end mill during the finishing process of a thin-walled aluminum alloy shell has practical technical value.

## Introduction

The degree development of the manufacturing industry reflects the comprehensive strength of the country, and plays a pivotal role in the high-speed development of the country's economic level, scientific , technological strength, and military power^1^. Nowadays, many countries have proposed development strategies and plans for the development of manufacturing industry. The United States has promulgated the "National Advanced Strategic Plan", the European Union has proposed "Strong EU Industry is Conducive to Economic Growth and Recovery", Japan has released the "White Paper on Manufacturing", and China has formulated the "Made in China 2025"^2^.

Aluminum alloy materials have been very widely used in aerospace small engines, large ship connectors, automotive transmission intermediate shell and other fields^3^. Because of its excellent properties such as small density, light weight, high strength, high temperature resistance, strong corrosion resistance, and easy assembly^4^. A high-quality toughening alloy created by heat treatment pre-tensile process, 6061 aluminum alloy is one of the many series of aluminum alloys. It has the advantages listed above as well as good formability, weldability, and excellent machinability^5^. It is widely used in the middle shell of the transmission with specific precision requirements and high corrosion resistance^6^. 6061 Aluminum alloy is a common material that is challenging to manufacture. It is simple to create tool sticking phenomena during the machining process, which can compromise the workpiece's surface precision and integrity^7^.

**Figure 1:**
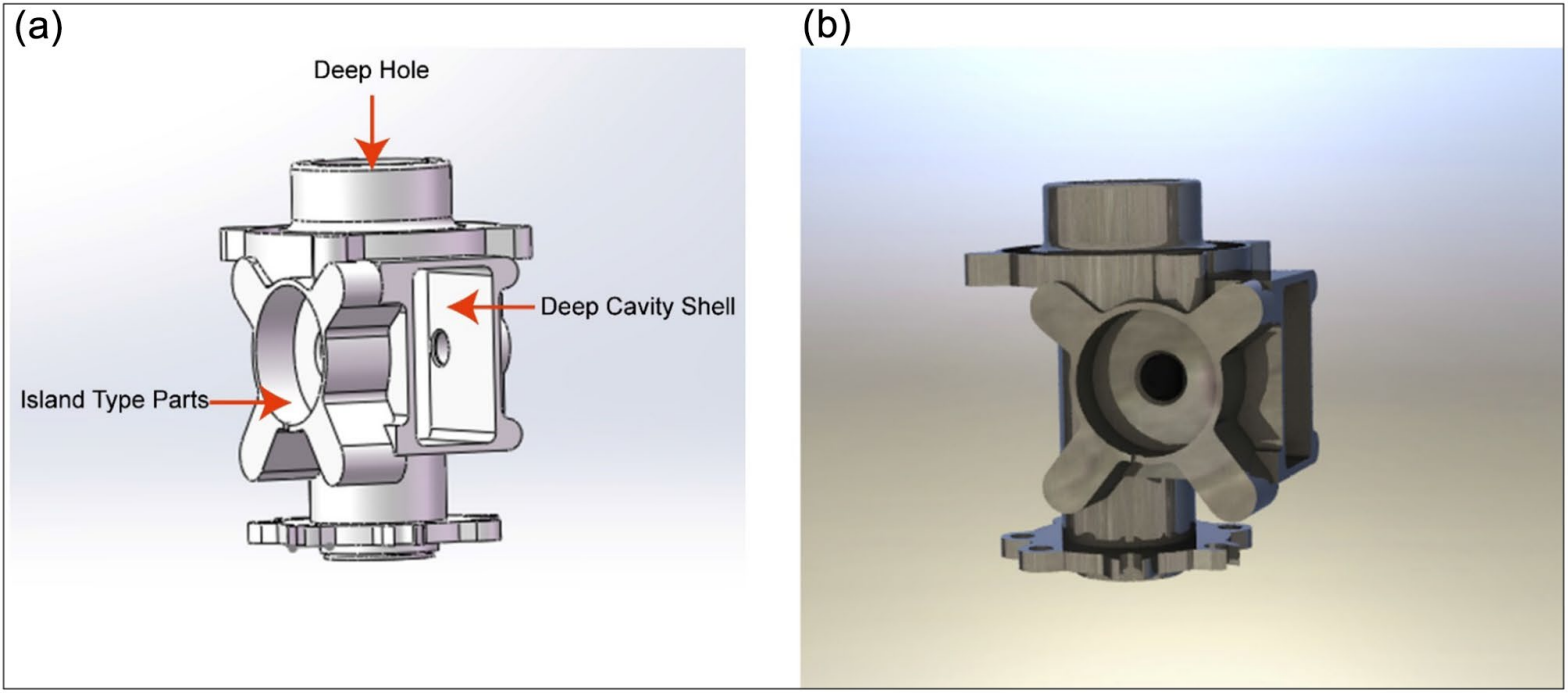
Transmission intermediate shell. (**a**) 3D modeling (**b**) Rendering models.

The transmission intermediate shell in Fig. 1 is widely used in automobile gearboxes. This part is an example of a thin-walled box or thin-walled shell. Its morphological features include several deep cavities, holes, and islands, all with complex geometries and difficult processing. There are many problems, such as high standards for precision, a quick rate of material removal, weak rigidity, and straightforward deformation of the workpiece^8^.

The machining process is quite challenging for this type of thin-walled shells with challenging-to-machine materials and intricately formed surfaces, and the issues of low efficiency and high cost are very critical. In order to solve and optimize engineering problems, cutting aluminum alloy shells has always been a critical need for relevant scholars^9^. Omar Fergani^10^ studied the milling problem of aluminum alloy reducer shell with deep cavity components, established a mathematical model based on predicting machining deformation, and verified the accuracy of the model through milling experiments, achieving the purpose of stable milling. Xu^11^ studied the automotive key components reducer shell, combined with high-precision composite machine tools, precision milling of deep holes, reducing the number of times of clamping and positioning, to achieve the overall production system efficiency, optimize the working environment and economic benefits. Song^12^ studied the thin-walled parts of high-temperature alloy magazine, by adopting the method of experimentally testing the processing status of the workpiece, comprehensively considering changes in spindle speed, axial depth of cut and tool position, a three-dimensional stable lobe diagram of the cutting process was established. , to realize the prediction of modal parameters in the cutting process. Dombovari^13^ studied the milling simulation system for thin-walled parts, through data collection, analysis and noise reduction processing of the cutting process, realizing the stability and feasibility prediction of milling and turning machining methods for thin-walled parts of the magazine by developing a complete milling simulation optimization system. Liu^14^ studied thin-walled titanium alloy compressor blades, he used the micro-milling machine tool Kern MMP and UG/CAM post-processing CNC programming to plan the tool path for the finishing process of the tiny impeller blades. Finally, he used a 1 mm diameter Carbide ball end milling cutter enables high-precision machining of the entire blade. Jafarian^15^ conducted a detailed study on the cutting parameters of nickel-based alloy aerospace thin-walled parts, by establishing a prediction model about the processing quality, an artificial bee colony algorithm was used to optimize the cutting parameters, and the accuracy of the model was verified through cutting experiments. Marques^16^ studied high-temperature alloy ship engine shells, innovatively used ceramic tools (Al2O3 + Si CW) for cutting high-temperature alloys, observed the flank wear of ceramic tools, analyzed the wear mechanism, and determined the optimal method for processing high-temperature alloy shells. In conclusion, according to the references^9–16^, numerous researchers have created mathematical models of cutting force or cutting temperature, and optimization algorithms to perform high-precision and high-quality machining of shells with complex profiles and difficult-to-machine materials. However, As the teeth of the manufacturing industry, metal cutting tools determine the surface accuracy and production cycle of the workpiece to be processed^17^, and the side milling method of integral end mills is widely used in the finishing process of thin-walled parts shells. Reasonable selection of integral end milling cutter side flute parameters can significantly improve the cutting efficiency and tool life^18^. At present, there aren't many studies on integral end mills' side edge optimization by simulation, experiment and machine learning intelligent algorithm comprehensive consideration, either domestically or internationally. As a result, the study of integral end mills' side edge optimization for completing aluminum alloy thin-walled part shells has engineering significance.

Based on the fundamentals of metal cutting, this article employs finite element simulation technology to convert a three-dimensional cutting process into a two-dimensional cutting process; it also studies the effects of tool parameters on cutting performance using orthogonal experimental methods and range analysis methods; The experiment carried out roughness detection on the workpiece, confirmed the accuracy of the finite element model and the validity of the orthogonal test, established a cutting force and roughness prediction model, and further optimized the tool parameters using the firefly algorithm. To get the best tool parameters design for the integral end mill for finishing thin-walled aluminum alloy parts.

### Simulation study of 6061 aluminum alloy milling process based on DEFORM-3D

The finite element analysis approach is widely employed in the field of advanced manufacturing as a result of the quick development of computer science and engineering. Among them, the finite element simulation software DEFORM-3D can analyze the coupling effects of various related physical fields during the metal forming process^19^, and also can carry out the simulation analysis of modeling, thermal conductivity, forging, and so on^20^. In the post-processing module^21^, several tendencies in physical quantity fluctuation can be seen. The ability to mesh intricate geometric models is DEFORM-3D's main benefit, after each stage, the model will be re-meshed^22^. And its own material library can provide a variety of metal and non-metal materials, demonstrating strong viability and dependability in the field of metal cutting^23^.

### Establishment of finite element simulation model

The environment variable parameters are specified as a single variable in order to have a good comparison and point of reference between the simulation and the physical experiment. It is assumed that the machine tool, fixture, and tool are all rigid bodies and that the ambient temperature is always 20℃^24^.

The impacts of temperature, material deformation, and stress–strain rate on the cutting process must be taken into account when establishing the model. The plastic deformation of materials under high strain rates is characterized in this article using the flow stress model Johnson–Cook constitutive model^25^.

1 $$\tilde{\sigma }=\left(A+B{\varepsilon }^{n}\right)(1+Cln\frac{\dot{\varepsilon }}{{\varepsilon }_{0}})\left[1-{(\frac{T-{T}_{amb}}{{T}_{melt}-{T}_{amb}})}^{m}\right]$$

In the Eq. (1), *A*, *B*, *n*, *C* and *m* are the material property strengthening term constants; *T*_*melt*_ is the melting point; *T*_*amb*_ is the room temperature; $\varepsilon$ is the reference strain rate; the primary alloying elements of 6061 aluminum alloy are silicon and magnesium, which together form the Mg_2_Si phase. The Mg_2_Si phase's chemical composition of chromium and manganese can counteract the negative effects of iron. A small addition of copper or zinc can enhance the alloy's strength, resistance to corrosion, and electrical conductivity^26^. The chemical compositions materials', mechanical and physical properties are displayed at the Tables 1, 2. Consulting the literature^27^ 6061 aluminum alloy Johnson–Cook constitutive model is:

**Table 1:** Chemical compositions (Wt.%) of AA 6061^28^.

Element	Al	Mg	Si	Zn	Cr	Mn	Fe	Ni	Cu	Ga
Wt.%	98.08	0.51	0.63	0.06	0.06	0.02	0.30	0.02	0.31	0.01

**Table 2:** Mechanical and physical properties of AA 6061^29,30^.

Material	Density (kg/m^3^)	Poisson ratio	Elastic module (MPa)	Melting point (ºC)	Specific heat (J/kg·K)	Thermal conductivity (W/m·K)	Yield strength (MPa)	Ultimate tensile strength (MPa)	Fracture strength (MPa)	Hardness (HV_0.1_)	Percentage elongation (%)
AA6061	2730	0.33	68,900	580–680	896	167	276	310	489	107	18%

2 $$\tilde{\sigma }=\left(324+114{\varepsilon }^{0.42}\right)(1+0.002ln\frac{\dot{\varepsilon }}{{\varepsilon }_{0}})\left[1-{(\frac{T-{T}_{amb}}{{T}_{melt}-{T}_{amb}})}^{1.34}\right]$$

With the progress of the cutting process, the changes in stress, strain and temperature are mainly concentrated in the stage of chip separation from the workpiece. At this stage, the internal physical properties of the material being processed change. The Johnson–Cook failure model^31^ is more accurate in the separation and characterization of aluminum alloy flocculated chips:

3 $$\overline{{\varepsilon }_{f}^{pl}}=\left[{d}_{1}+{d}_{2}{exp({d}_{3}\frac{p}{q})}^{n}\right]\left[1+{d}_{4}{\text{ln}}(\frac{{\dot{\overline{\varepsilon }}}^{pl}}{\dot{{\varepsilon }_{0}}})\right]\left(1+{d}_{5}\widehat{\theta }\right)$$

In the Eq. (3), *d*_1_, *d*_2_, *d*_3_, *d*_4_, *d*_5_ are failure parameters under conditions below the transformation temperature; $\dot{{\varepsilon }_{0}}$ is the reference strain rate; ${\dot{\overline{\varepsilon }}}^{pl}$ is the plastic strain rate; consult the literature^32^ Johnson–Cook shear failure model of 6061 aluminum alloy is as follows:

4 $$\overline{{\varepsilon }_{f}^{pl}}=\left[0.071+1.248{exp(-1.142\frac{p}{q})}^{n}\right]\left[1+0.147{\text{ln}}(\frac{{\dot{\overline{\varepsilon }}}^{pl}}{\dot{{\varepsilon }_{0}}})\right]\left(1+1.87\widehat{\theta }\right)$$

Wear will occur in the continuous cutting process, and the Usui model can better reflect the metal cutting process^33^.

5 $$\upomega =\int {apve}^{-b/T}{\text{dt}}$$

**Figure 2:**
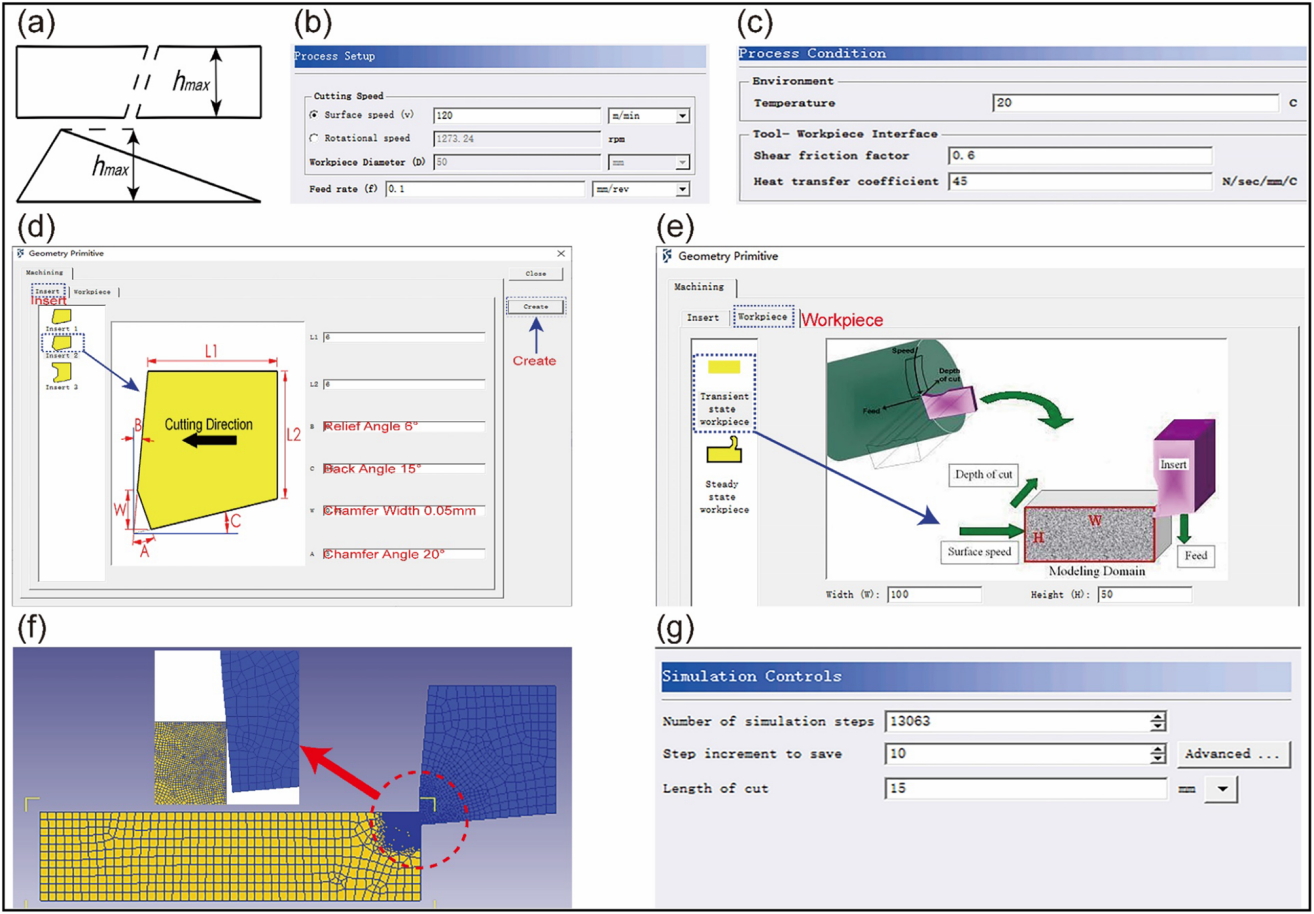
Cutting simulation settings based on DEFORM-3D (**a**) Equivalent cutting thickness model (**b**) Setting of cutting parameters (**c**) Setting of environmental variables (**d**) Setting of tool parameters (**e**) Setting of workpiece parameters (**f**) Meshing (**g**) Simulation control.

In the Eq. (5), *ω* is the wear depth; *p* is the contact pressure; *v* is the sliding speed; T is the temperature; a = 0.000001; In the actual milling process, the cutting part of the milling cutter is generally used for cutting, which is approximately an external turning tool and can be studied by referring to the turning principle^34^. The chip thickness changes continuously and uniformly while the milling cutter rotates in addition to executing a feeding operation, as shown in Fig. 2a. The cutting parameters and environmental factors are established using a combination of the metal cutting criteria and the currently in use processing circumstances, as shown in Fig. 2b, c. The tool parameters (chamfer width, chamfer angle, rake angle, relief angle) and workpiece (length, height) were precisely established in order to analyze the physical properties of the milling cutter side edge cutting operation. Taking one set of experiments as an example, Fig. 2d illustrates the reasonable range of tool parameters for finishing 6061 aluminum alloy: chamfer width 0.05–0.2 mm, chamfer angle 5°–20°, rake angle 6°–15°, and relief angle 18°–24°. Workpiece parameters: length 100 mm, height 50 mm, as shown in Fig. 2e. The term "tool material" refers to WC-based cemented carbide with a TiN coating on the surface. The workpiece material is defined as 6061 aluminum alloy. Figure 2f illustrates the meshing of the tool and workpiece. Lastly, according to the instructions in Fig. 2g, setting the number of simulation steps, validating the data, and creating the BD. file. The post-processor module can be opened to view the cutting condition of each step after the cutting process has finished.

### Post-processing data analysis

**Figure 3:**
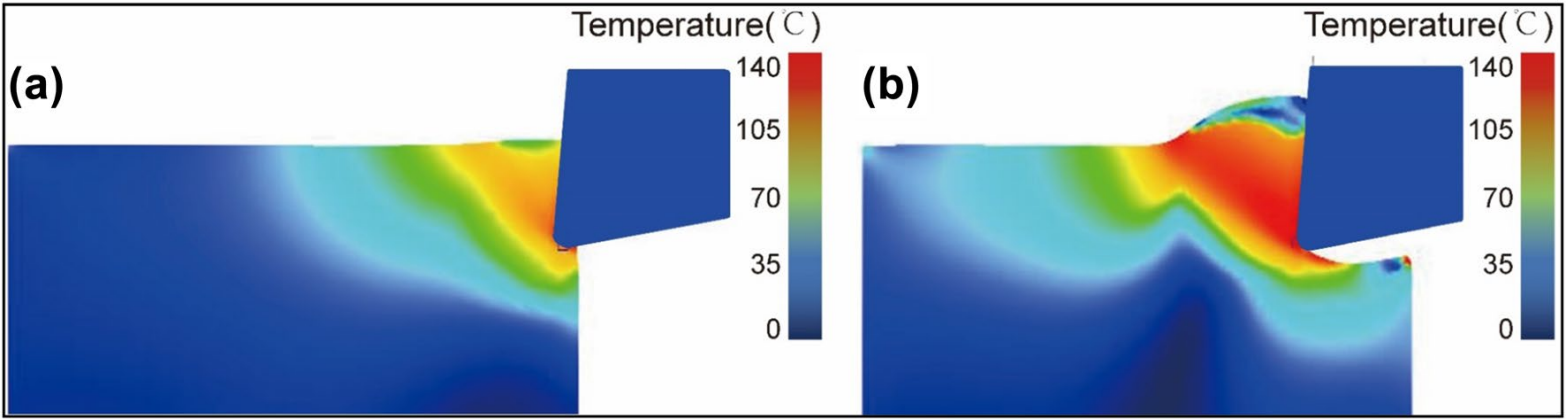
Distribution cloud diagram of cutting temperature (**a**) Starting cutting stage (**b**) Stable cutting stage.

Metal cutting is a material removal operation that generates a significant amount of cutting force and cutting heat. Cutting force and cutting heat are significant evaluation factors that characterize the milling process, which frequently impacts the wear and service life of the tool and has an effect on the workpiece's surface roughness^35^. The temperature distribution cloud diagram at the beginning and stable stages of cutting are shown in Fig. 3.

**Figure 4:**
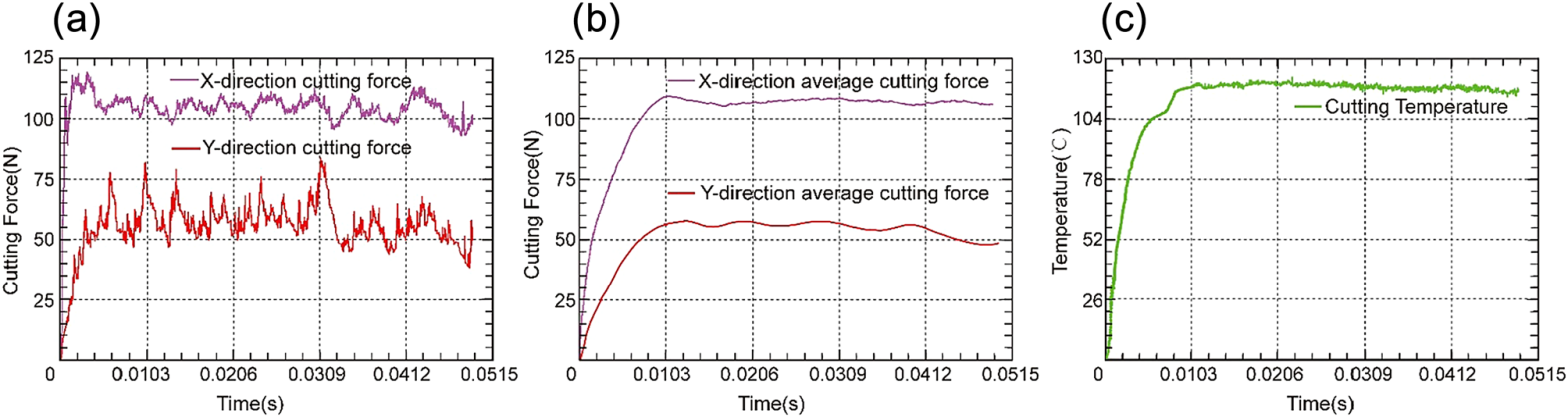
Post-processing results (**a**) Cutting force in X and Y directions (**b**) Average cutting force in X and Y directions (**c**) Cutting temperature.

The cutting forces generated during the process of simulation can be shown as periodic wave form graphs in the DEFORM-3D post-processing output. The cutting force in the X direction and the cutting force in the Y direction change over time, as shown in Fig. 4a. When cutting into the workpiece, the tool is in an unstable condition. The cutting force gradually grows from zero as the cutting process progresses, without any evident dramatic changes, and tends to be stable. After the data has been processed, the average cutting forces in the X and Y directions are identified, according to Fig. 4b. Figure 4c shows the evolution of the cutting temperature trend over time. The cutting temperature steadily rises from zero as the cutting process continues, without any obvious mutation, and has a tendency to remain steady.

### Orthogonal test and range analysis

The orthogonal test method uses a series of properly ordered orthogonal tables to conduct extensive comparisons, extensive analysis, and statistical calculations, which considerably lowers the cost of testing under multi-factor situations by reducing the number of tests^36^.

The Ll6 (4^4^) four-factor four-level orthogonal test was employed to examine the effects of milling cutter chamfer width and chamfer angle, rake angle, and relief angle on the cutting force and cutting temperature in order to better understand the influence of milling cutter parameters on cutting performance. The data are processed, the results are calculated, and the range analysis is done for the orthogonal test to produce the orthogonal test Table 3.

**Table 3:** Orthogonal test.

Test number	Chamfer width *b*_*01*_ (mm)	Chamfer angle *r*_*01*_ (°)	Rake angle γ_0_ (°)	Relief angle *α*_*0*_ (°)	Average cutting force/F (N)	Average cutting temperature/T (℃)
1	1 (0.05 mm)	1 (-20°)	1 (6°)	1 (15°)	105.6	128.5
2	1	2 (-15°)	2 (9°)	2 (18°)	118.7	112.3
3	1	3 (-10°)	3 (12°)	3 (21°)	109.2	125.8
4	1	4 (-5°)	4 (15°)	4 (24°)	118.7	95.3
5	2 (0.10 mm)	1	2	3	121.5	132.6
6	2	2	3	4	130.3	124.5
7	2	3	4	1	106.2	114.6
8	2	4	1	2	113.1	121.3
9	3 (0.15 mm)	1	3	1	128.8	128.9
10	3	2	4	2	90.7	110.3
11	3	3	1	3	108.8	101.5
12	3	4	2	4	90.6	89.5
13	4 (0.20 mm)	1	4	3	135.3	133.9
14	4	2	1	4	121.1	134.3
15	4	3	2	1	117.2	111.2
16	4	4	3	2	107.4	130.1

The range analysis results in Table 4 lead to the following conclusion: R_A_ > R_B_ > R_D_ > R_C_, meaning that the cutting force is most significantly impacted by the chamfer width, then by the chamfer angle, then by the relief angle, and least significantly by the rake angle.

**Table 4:** Range analysis of cutting force.

	A (*b*_*01*_)	B (*r*_*01*_)	C (γ_0_)	D (*α*_*0*_)
I	113.1	122.8	112.2	114.5
II	118.8	115.2	112.0	107.5
III	104.7	110.3	118.9	118.7
IV	120.3	107.4	112.7	115.2
R	15.5	15.4	6.9	11.2
Factor priority	ABDC			

The range analysis results in Table 5 lead to the following conclusion: R_B_ > R_A_ > R_C_ > R_D_, meaning that the cutting temperature is most significantly impacted by the chamfering angle, then by the chamfer width, then by the rake angle, and least significantly by the relief angle.

**Table 5:** Range analysis of cutting temperature.

	A (*b*_*01*_)	B (*r*_*01*_)	C (γ_0_)	D (*α*_*0*_)
I	115.5	131.0	121.4	120.8
II	123.3	120.4	111.4	118.5
III	107.6	113.3	127.3	123.4
IV	127.4	109.1	113.5	110.9
R	19.8	21.9	15.9	12.5
Factor priority	BACD			

**Figure 5:**
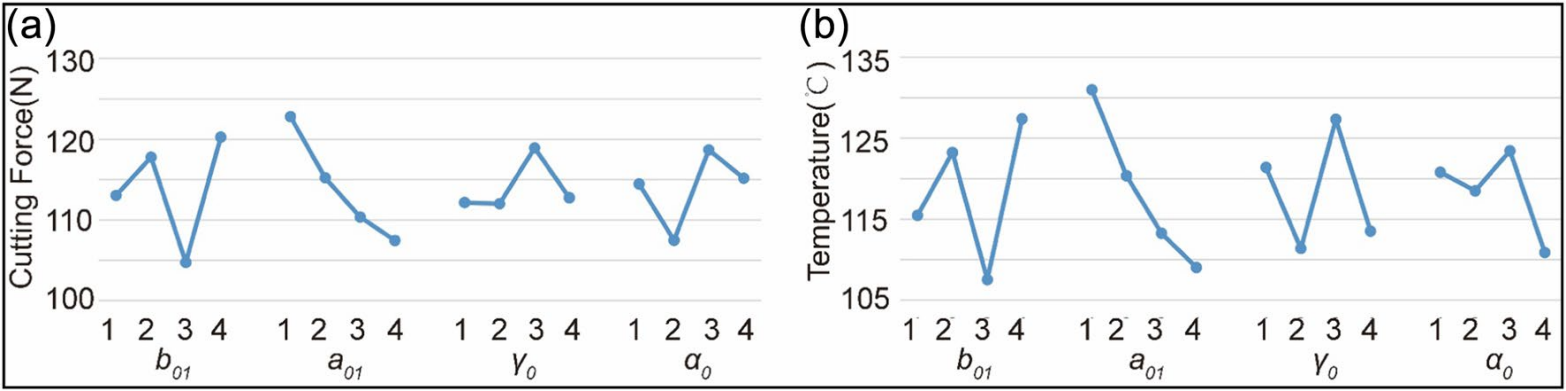
Range analysis (**a**) Range analysis of cutting force (**b**) Range analysis of cutting temperature.

According to Fig. 5, it can be further seen that the impact of tool chamfering on cutting performance is greater than that of tool rake angle and relief angle, accounting for 63.1% and 59.5% respectively. Because it controls the area of contact between the side edge and the workpiece, the chamfer width is the primary determinant of cutting force. As the chamfer width increases, the cutting force first increases, then decreases, and then increases. However, the overall cutting force increases with the increase of the chamfer width. The cutting force will decrease as the chamfer width increases from 0.1 to 0.15 mm. In conclusion, the cutting force decreases and the point since the chamfer width is bigger than the feed rate. However, when the chamfering width continues to increase, the contact area increases between the tool side edge and the workpiece, and the tool surface will adhere to a laminated chip bump, which indirectly increases the cutting force. The chamfer angle is a secondary influencing factor of cutting chips can be released more smoothly at this force. The cutting force increases as the chamfer angle increases, and the increase becomes more obvious. The chamfer angle, however, cannot always be increased. The cutting edge becomes tougher as the chamfer angle increases. The tool is more prone to wear and chipping the thinner it is. In terms of decreasing the cutting force of the tools, the best design parameters combination is A3B4C2D2. In the same way that chamfer width affects cutting force, it also has an impact on cutting temperature. However, the key element affecting cutting temperature is the chamfer angle. The cutting edge will be sharper and the tool's overall heat dissipation performance will be worse as the chamfer angle increases. As the cutting temperature rises, tool wear accelerates. Choosing a chamfer angle wisely can significantly increase tool service life. In terms of decreasing the cutting temperature, the best design parameters combination is A3B4C2D4. Overall, when side milling thin-walled aluminum alloy parts, the cutting edge geometry has a significant impact on the cutting performance.

### Cutting experiment and result analysis

The workpiece surface roughness was assessed, and 16 sets of cutting tests were carried out to confirm the simulation results and to validate the correctness of the finite element model and the validity of the orthogonal test method.

### Cutting experiment

**Figure 6:**
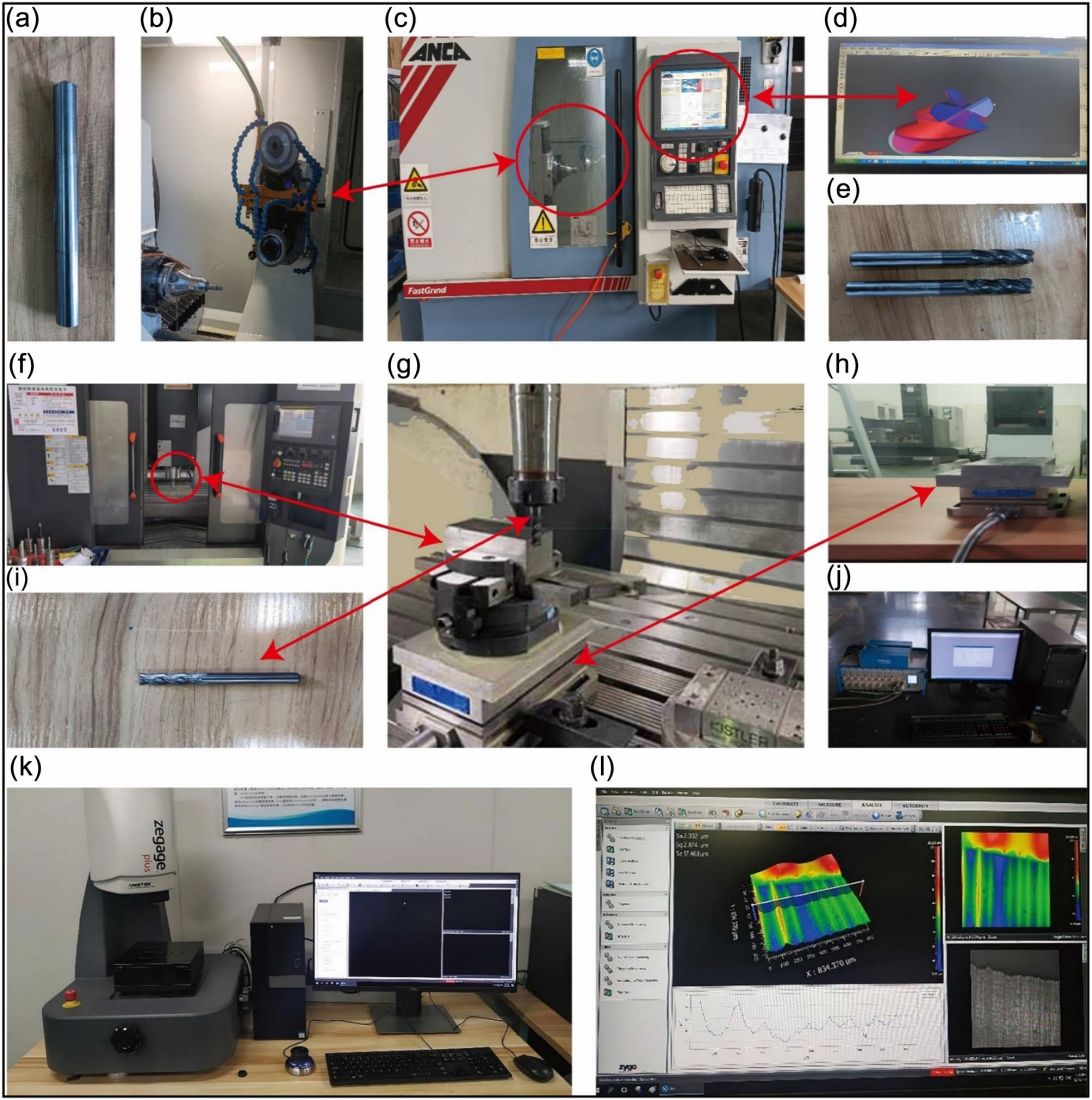
Experimental equipment (**a**) Milling cutter bar (**b**) Grinder grinding device (**c**) ANCA TGX universal grinder (**d**) CIMulator3D module (**e**) Preparing milling cutter (**f**) VMC850B vertical machining center (**g**) Cutting processing device (**h**) Swiss Kistler9225B three-way measuring instrument (**i**) Experimental milling cutter (**j**) Yokogawa MX100 thermocouple thermometer (**k**) AMETEK zygo interferometer (**l**) Surface roughness measurement and calculation.

Due to the different tool parameters required for each cutting experiment, in order to reduce the procurement cost, ANCA TGX universal grinding machine is used to grind the tool, and CIMulator3D module is used to view the grinding tool process. The cutting experiment was carried out on the VMC850B vertical machining center, and the cutting force and cutting heat data acquisition system was established. The final physical experiment data was determined to be the average value of the data gathered. When measuring the workpiece's surface roughness, the surface roughness is measured five times equally separated, and the average value is taken after the maximum and minimum values are subtracted. Details of cutting parameters and other experimental equipment are shown in Table 6, and experimental equipment is shown in Fig. 6.

**Table 6:** Experimental parameters and equipment details.

Tool parameters	Workpiece parameters	Cutting parameters	Machine information	Data collection system	Roughness detection system
WC-based carbide TiN coated four-edge end mill, D = 8 mm, β = 30°	Al6061-T6 cuboid blank, L = 70 mm, W = 40 mm, H = 20 mm	*a*_*p*_ = 0.5 mm, *v*_*c*_ = 120 m/min, *f* = 0.1 mm/r	ANCA TGX Universal Grinder, Fig. 6c, VMC850B Vertical Machining center, Fig. 6g	Kistler9225B three-way measuring instrument, Swiss, Fig. 6h, Yokogawa MX100 Thermocouple Thermometer, Fig. 6j	AMETEK Zygo Interferometer, Fig. 6k

### Analysis of experimental results

**Figure 7:**
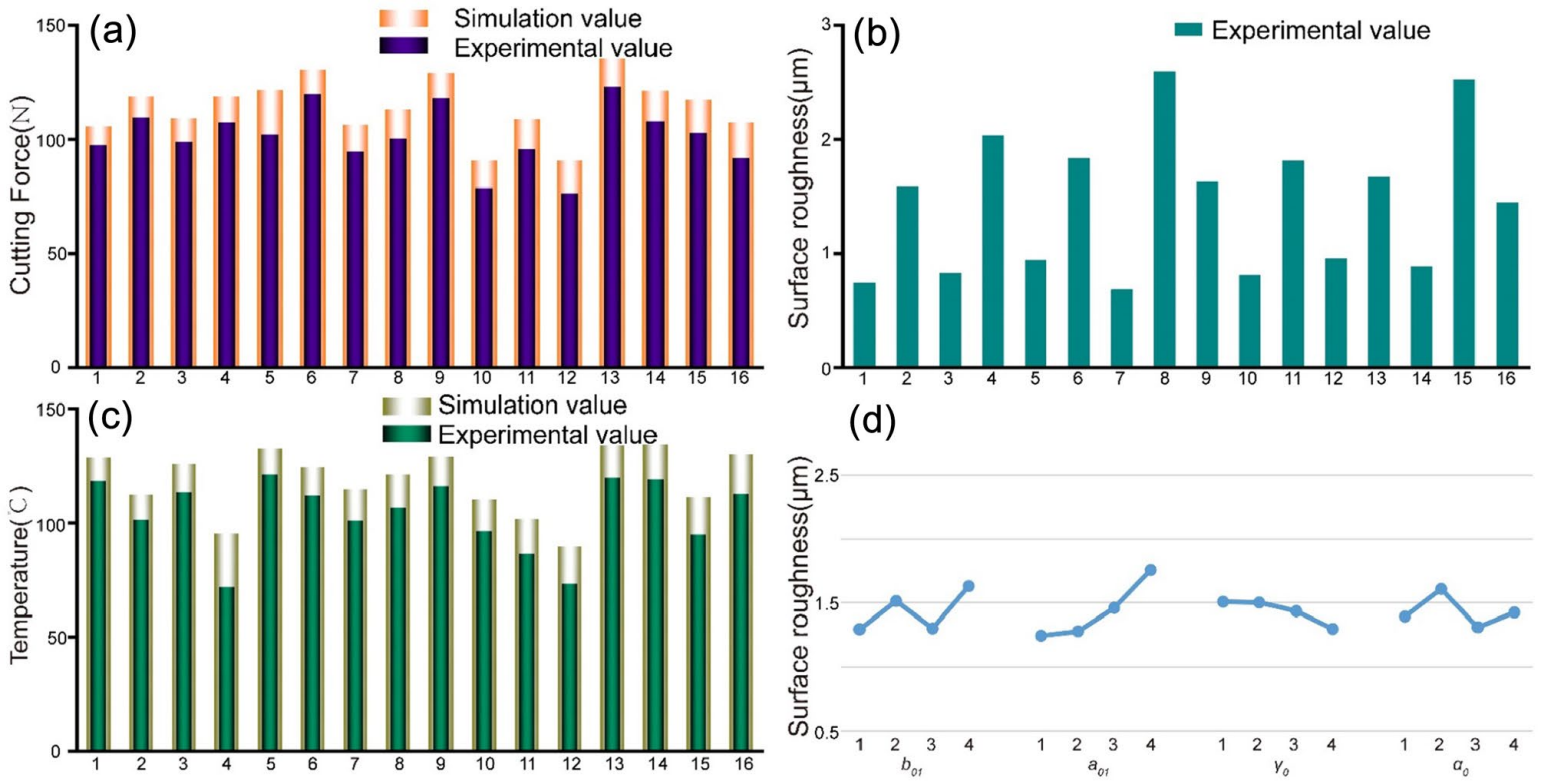
Data analysis (**a**) Comparison of cutting force simulation values and experimental values (**b**) Experimental values of surface roughness (**c**) Comparison of cutting temperature simulation values and experimental values (**d**) range analysis of surface roughness.

It can be seen from Fig. 7a, c that the results obtained by the physical cutting experiment and the data obtained by the finite element simulation program DEFORM-3D have similar variation trends, which indicates that the established finite element simulation model is accurate and the orthogonal test method is reasonable. By analyzing the data, it can be shown that the fifth group of tests' cutting force simulation value and experimental value differ by up to 16%, so the experimental value of cutting force can reflect the accuracy of the simulation value. In the fourth group of experiments, the difference between the experimental value of cutting temperature and the simulation value is up to 24%, which is slightly higher, but the average difference between the experimental value and the simulation value of the whole 16 groups of experimental data is 12.6%, so the cutting temperature can still reflect the accuracy of the simulation value. Among them, the experimental values of cutting force and cutting temperature are smaller than the simulation values, and the main reasons for this phenomenon can be attributed to: the finite element simulation cutting environment cannot completely simulate reality. The metal cutting process is a continuous and dynamic thermal coupling process. When the tool squeezes the workpiece, the cutting area will instantly generate cutting force and cutting heat. With the cutting process, the vibration of the machine tool spindle, the thermal softening characteristics of the workpiece, and the plasticity of the material, flow and environmental heat exchange effects will affect the changes in cutting force and cutting heat. At the same time, when the simulation model was set up in DEFORM-3D in the early time, taking into account factors such as the calculating power of the computer, the stability and sustainability of the simulation environment, and time cost, the simulation model ignored system vibration, wear, and complex thermal coupling processes. Which will have an impact on the force and heat changes in the cutting process. But the simulation model can be used for tool parameters optimization overall.

During the cutting process, surface roughness is a key indicator to measure the quality of the machined surface and the quality of the machining process^37^. According to the range analysis results in Table 7, it can be concluded that: R_B_ > R_A_ > R_D_ > R_C_, that is, the chamfer angle has the greatest impact on roughness, followed by the chamfer width, then the relief angle, and the rake angle has the least impact on the roughness. Figure 7b shows the test value of surface roughness after cutting. The eighth group of experiments shows the maximum roughness of 2.59μm, which meets the accuracy requirements of the workpiece. Figure 7d shows the range analysis of roughness test values. To sum up, the influence of tool chamfering on surface roughness is still greater than that of tool rake angle and relief angle, accounting for 62.6%. The surface roughness increases first, then decreases and then increases with the increase of chamfering width, which is the same as the cutting force and cutting temperature with the change of chamfering. When the chamfering width (0.05 mm, 0.15 mm) is less than or slightly larger than the feed rate, the roughness is relatively low and the surface quality is better. The reasons are as follows: when the chamfering width is 0.05 mm, the cutting vibration is lower and the roughness is smaller. When the chamfering width is 0.10 mm, the cutting vibration increases and the roughness increases. When the chamfer width (0.15 mm) is slightly larger than the feed rate, the chips can be discharged more smoothly, the cutting edge plays a smoothing role, and the roughness decreases; However, when the chamfer width (0.20 mm) continues to increase, the contact area between the tool side edge and the workpiece become larger, and the cutting vibration increases, so does roughness. The surface roughness increases with the decrease of the chamfer angle, and the increase becomes more and more obvious. When the chamfer angle decreases from 20° to 15°, the side edge of the tool becomes slightly blunt, and the roughness increases slightly at this time. When the chamfering angle continues to decrease, the contact area between the tool side edge and the workpiece increases, the material removal rate increases, the aluminum alloy sticking tool effect gradually appears, and the roughness increases. However, it does not mean that the larger chamfering angle, the better cutting effect, when the chamfering angle is too large, the tool side edge is sharp, and it is easy to produce a collapsing edge phenomenon, which will increase the surface roughness.

**Table 7:** Range analysis of surface roughness.

	A (*b*_*01*_)	B (*r*_*01*_)	C (γ_0_)	D (*α*_*0*_)
I	1.297	1.246	1.508	1.395
II	1.514	1.279	1.502	1.609
III	1.303	1.462	1.435	1.313
IV	1.63	1.757	1.299	1.427
R	0.333	0.511	0.208	0.296
Factor priority	BADC			

### Establishment of prediction model and optimization of tool parameters

Cutting experiments have verified the accuracy of the finite element model and the rationality of the orthogonal test method. On the basis of the currently available experimental data, a surface roughness prediction model was created in order to further control, tweak, and optimize the surface roughness process parameters^38^.

### Establishment of prediction model

The empirical formula prediction model is another name for the exponential function-based prediction model. It is frequently utilized in the surface roughness, cutting force, and cutting temperature prediction field^39^ since the mathematical model can only be developed by a large quantity of test data, and aspects like the cutting mechanism and metal material separation criteria need not be explicitly taken into account. The empirical formula is as follows:

6 $$\theta =K{ b}_{01}^{{M}_{1}} {r}_{01}^{{M}_{2}}{ \gamma }_{0}^{{M}_{3}} {\alpha }_{0}^{{M}_{4}}$$

In the Eq. (6): *K*- Influence coefficient. Taking the logarithm of both sides of Eq. (6) and transforming it into a multiple linear regression equation:

7 $$ln\theta =lnK+{M}_{1}ln{b}_{01}+{M}_{2}ln{r}_{01}+{{M}_{3}ln\gamma }_{0}+{{M}_{4}ln\alpha }_{0}$$

Setting up:

8 $$y=\mathit{ln}\theta ,{M}_{0}=lnK{,x}_{1}=ln{b}_{01}{,x}_{2}=ln{r}_{01}{,x}_{3}={ln\gamma }_{0},{x}_{4}={ln\alpha }_{0}$$

Then Eq. (7) is converted to:

9 $$y={M}_{0}+{M}_{1}{x}_{1}+{M}_{2}{x}_{2}+{M}_{3}{x}_{3}+{M}_{4}{x}_{4}$$

The regress () function in MATLAB are was used to calculate and process 16 groups of data, and the linear regression equation of the roughness prediction model was obtained^40^:

10 $${R}_{a}=2.904{ b}_{01}^{0.136} {r}_{01}^{-0.251}{ \gamma }_{0}^{-0.061} {\alpha }_{0}^{-0.102}$$

Regression analysis and *F* test were performed on the roughness prediction model, *F* = 91.22 > *F*_0.05_(4,11) = 5.936, and the regression equation of the prediction model was highly significant at the level of α = 0.05, indicating that the surface roughness formula and experimental data fit each other with high reliability.

In the roughness prediction model, the index of the tool chamfer is greater than the index of the tool rake angle and relief angle, indicating that the impact of the tool chamfer on roughness is greater than the tool rake angle and relief angle; and the index of the chamfer angle is the largest, the index of the rake angle is the minimum, indicating that the chamfer angle has the greatest impact on roughness, and the rake angle has the least impact on roughness. This is consistent with the conclusion drawn from the roughness range analysis, which illustrates the accuracy, effectiveness and rationality of the roughness prediction model once again.

In the same way, the linear regression equation of the cutting force and temperature prediction model was derived after 16 groups of experimental data were computed and processed. The formula as follows:

11 $$F=62.9{ b}_{01}^{15.1} {r}_{01}^{1.15}{ \gamma }_{0}^{0.93} {\alpha }_{0}^{0.322}$$

12 $$T=98.0{ b}_{01}^{5.34} {r}_{01}^{14.58}{ \gamma }_{0}^{0.033} {\alpha }_{0}^{0.842}$$

### Tool parameters optimization based on firefly algorithm

Generally speaking, in the case of determining the cutting parameters, a reasonable choice of tool parameters can greatly reduce the surface roughness. In order to more accurately predict the effects of tool chamfer, rake angle, and relief angle on roughness and cutting force. It is required to further refine the provided prediction model in order to make it more optimal^41^.

Firefly algorithm is a heuristic bionic swarm intelligent optimization algorithm inspired by the information exchange of natural fireflies through fluorescence^42^. Fireflies attract each other through their own brightness and attraction, and are inversely proportional to their distance. The brightness of their fluorescence depends on the target value of their location. The better the location, the better the target value^43^.

**Figure 8:**
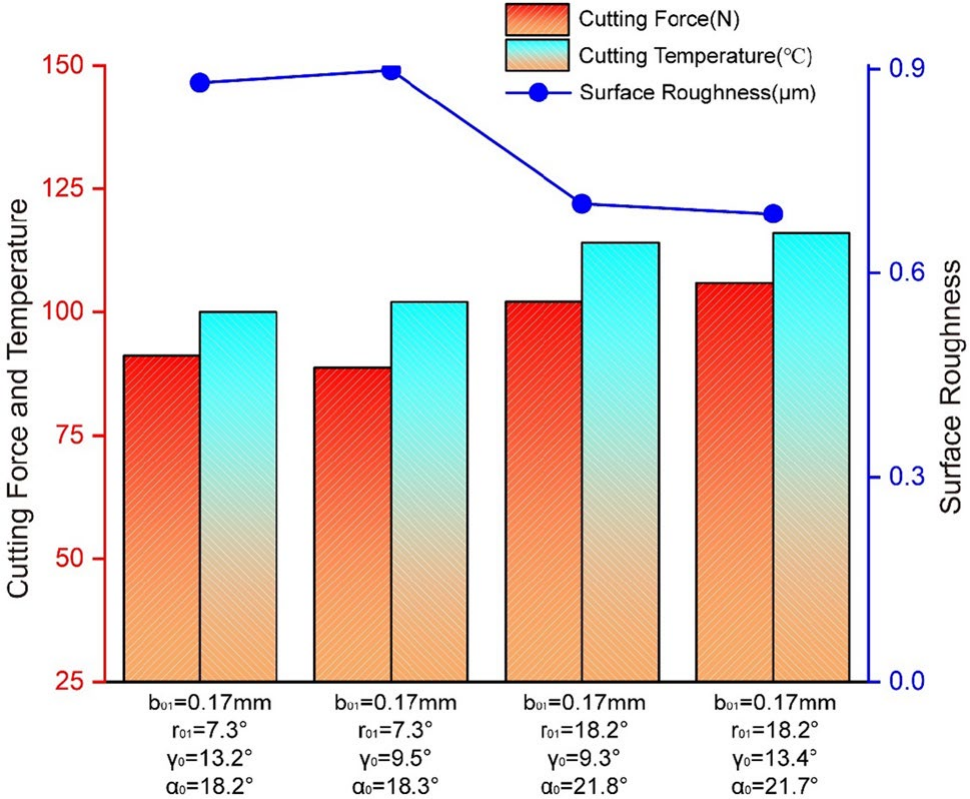
Typical data for tool parameters optimization.

Since the firefly algorithm is to find the position of the strongest brightness, the purpose of this study is to find the geometric parameters of the tool that make the cutting force minimum and the roughness minimum. MATLAB software was used to carry out dual-objective optimization, firefly algorithm in the algorithm toolbox was called, and various parameters were set^44^. In order to avoid overtraining in the algorithm iteration process, the number of iterations is set to 2000, the initial population number is 200, and the constraint conditions for tool parameters optimization are as follows: *b*_*01*_ = [0.05,0.20], *r*_*01*_ = [-20,-5], *γ*_*0*_ = [6, 15], *α*_*0*_ = [15.,24]. Typical data selection is shown in Fig. 8.

According to data analysis in Fig. 8:

1. The rake angle and relief angle should be taken in a variety of ranges, while the optimal chamfering width and chamfering angle should be taken in a specific range. The findings demonstrate that chamfering width and chamfering angle are the most significant influences on cutting force and roughness, whereas rake angle and relief angle have negligible effects. This is consistent with both the range analysis of cutting force and the range analysis of roughness's findings.
2. The range of the optimized tool's chamfering width, 0.15–0.17 mm, is comparable to the ranges found by analyses of the cutting force and roughness, and the optimized roughness value is lower than the unoptimized roughness value. The mathematical model of tool parameters optimization based on the firefly algorithm is given to show its rationale and feasibility.
3. The optimal chamfering angle varies between 18.2° below 20° and 7.3° above 5°. The optimized roughness value is lower than the unoptimized roughness value, the cutting force approximately increases with increasing chamfering angle, the cutting temperature approximately increases with increasing chamfering width, while the roughness approximately decreases with increasing chamfering angle. This is consistent with the conclusion obtained from the cutting force range analysis, cutting temperature range analysis and roughness range analysis. That illustrates the rationality and feasibility of the tool parameters optimization mathematical model based on the firefly algorithm once again.

## Conclusion

1. The cutting process of the aluminum alloy transmission intermediate shell has always been a research topic for many scholars. In this article, the three-dimensional mathematical model of the aluminum alloy shell is established firstly, and the two-dimensional cutting simulation physical model is established in the finite element software DEFORM-3D, and the orthogonal test method is designed.
2. Then, the data was calculated, processed, and range examined in the post-processing module to create the curve diagram of the influence of tool geometric parameters on cutting performance. It is determined that tool chamfering has a bigger impact on cutting performance than tool rake angle and relief angle. The primary determinant of cutting force is chamfering width, while the primary determinant of cutting temperature is chamfering angle. Aiming at lower cutting force and cutting heat, the optimal tool choice is A3B4C3D2 or A3B4C2D4.
3. Field cutting experiments were performed to validate the test scheme and measure the workpiece's surface roughness. It has been determined by data collecting, processing, and range analysis that the finite element simulation model can correctly and truthfully depict the physical experimental cutting process. Additionally, the tool's chamfering had a greater effect on roughness than the tool's rake and relief angles. Aiming at lower surface roughness, the optimal tool choice is A3B1C4D3.
4. Finally, the prediction model of cutting force, cutting force and roughness are built in order to further optimize the tool parameters, and it is iteratively improved by the firefly algorithm in the multi-objective optimization algorithm toolbox in MATLAB. The firefly method is found to be capable of optimizing the tool parameters through comparison analysis. The optimized tool parameters are *b*_01_ = 0.17 mm, *r*_01_ = 7.3° or *r*_01_ = 18.3°.

It is demonstrated that the optimization of the integrated end mill's side edge characteristics, which was utilized to finish a thin-walled aluminum alloy shell, has significant engineering applications.

## Data Availability

The data used to support the findings of this study are available from the corresponding author upon request.
